# Supplementary material for: Are carbon emissions trading and green financial instruments synergistic? -Comprehensive quantitative research based on content analysis
Source: PLoS One. 2024 Mar 7;19(3):e0298601. doi: 10.1371/journal.pone.0298601 (PMC10919601; doi:10.1371/journal.pone.0298601)
Supplement: S1 Data — (ZIP) [file pone.0298601.s001.zip › Data/地级市Z_F工作报告.zip!/║■─╧/│╗╓▌╩╨/└┤╫╘╣1⁄2╓┌║┼í╛┬φ┐╦╩2╛▌═°í┐_2014.docx]

各位代表：

现在，我代表市人民政府向大会报告工作，请予审议，并请市政协各位委员及其他列席人员提出意见。

一、2013年工作回顾

2013年是郴州科学发展、推动起飞迈出坚实步伐的一年。面对世界经济复苏艰难、国内经济增速放缓的严峻形势，全市上下在市委的坚强领导下，统筹稳增长、调结构、促改革、惠民生，较好地完成了市四届人大二次会议确定的年度目标任务。预计(下同)，地区生产总值1682亿元，同比增长11%;社会消费品零售总额625亿元，增长12.7%;财政总收入216.1亿元、公共财政预算收入146.5亿元，分别增长18.7%和22.4%;城乡居民人均可支配收入分别达19000元、8400元，分别增长9%和14%，农村居民收入增幅连续4年高于城镇居民;节能减排完成年度任务，万元GDP能耗下降4%。资兴、桂阳、永兴进入2012年度全省县域经济十强县(市)，北湖、宜章进入2012年度全省县域经济发展先进县市区，永兴入选全省首批特色县域经济重点县。

——项目建设强力推进。把项目建设作为稳增长、调结构、惠民生的主抓手，实施“十二五”投资和项目建设“836”计划，开展重点项目“百日攻坚”，完成固定资产投资1471亿元，增长34%。围绕交通大建设、产业大转型、城市大提质，实施投资1亿元以上主要建设项目779个，完成投资1082亿元，其中纳入省“三个一”行动计划的60个重大项目完成投资225亿元。交通大建设完成投资141亿元，衡茶吉铁路郴州段、炎汝高速公路、耒宜高速公路提质改造等建成通车，新建改造干线公路通车里程超过100公里。台达产业园二期、格兰博智能机器人、宜章氟化学工业园、安仁稻田公园、风电等275个产业项目，郴州国际会展中心、郴江路延伸段、骆仙铺片区城市道路及配套等200个城市基础设施项目，市体育中心体育馆、城东新区居民安置房、城镇垃圾处理设施等240个民生和社会事业项目竣工或完成年度计划。争取518个重大项目补充纳入中省“十二五”专项规划。东江引水工程、郴州机场、莽山水库等重大项目前期工作取得突破，桂永郴赣铁路列入国家中长期铁路网调规取得进展。各类融资245亿元，报批项目用地2.42万亩，较好地破解了资金、土地瓶颈。经济增长的内生动力增强。

——产业转型步伐加快。实施“产业转型发展三年行动计划”，三次产业结构调整为9.8：57.7：32.5，第三产业比重提高0.8个百分点。工业经济提质增效。扎实开展“改造提升传统产业年”活动，工业技改投资增长30%，新增规模以上工业企业82家，规模以上工业增加值增长12.5%、利润增长16%;省级园区规模以上工业增加值占全市规模以上工业的比重提高5个百分点。有色金属“五个一”战略体系和国家有色贵重金属产品质检中心建设加快推进，南方稀贵金属交易所通过国家验收。新认定国家高新技术企业15家，郴州高新区获批省科技成果转化示范基地，全市高新技术产业增加值增长35%。8件商标进入中国驰名商标评审。专利授权920件。关闭“六小企业”300多家。3个园区、6家企业分别列入全省循环经济试点示范园区和示范企业，资兴市入围国家循环经济示范城市，郴州获省低碳城市试点。现代服务业加快发展。旅游“五个一工程”和“中国温泉之城”建设顺利推进，2013年中国(郴州)国际休闲旅游文化节成功举办，宝山矿山公园、九龙江森林公园成功创建国家4A级景区，东江湖景区成功创建首批国家生态旅游示范区、创国家5A级景区近期可通过验收，桂东县获评中国最佳生态旅游示范县。接待游客人数、旅游总收入分别增长18.9%和21.2%。创建金融生态良好城市进展顺利，金融机构各项存贷款余额分别增长15%和19%，新增贷款110亿元;农村信用社改革深入推进，郴州农商行筹建上报银监会待批;新增小额贷款公司6家、融资性担保公司1家、省重点上市后备企业2家，发行企业债券39.5亿元。“市场建设三年行动计划”加快推进，完成标准化农贸市场改造40个，建成标准化农家店170家，美美世界、步步高新天地、友阿国际广场等7个城市综合体和万华汽车城、高壁综合大市场等9个专项市场建设进展顺利。公路水路客货运周转量、邮电业务量分别增长15%和8%。房地产开发投资、商品房销售面积分别增长28.7%和20%。农业现代化水平提升。克服历史罕见旱涝急转特大灾害影响，粮食总产量183万吨，收购烟叶110.1万担。改造中低产田和建设高标准农田10.5万亩，实施农业标准化面积240万亩。建成标准化养殖场(小区)50个，新扩蔬菜专业基地1.2万亩，新增果园15万亩、茶园1.5万亩。桂东县成为全国百强重点产茶县。推进现代庄园经济发展，创建全国休闲农业示范点1个、国家五星级农庄2家。桂阳县获评全国休闲农业与乡村旅游示范县。新增现代农业示范园区11个、农民专业合作社510家、省级农业龙头企业12家，培育年产值过5亿元企业2家，农产品加工业产值增长20%。永兴、宜章列入全国农产品产地初加工试点县。流转农村土地504.8万亩。推广农机具5.6万台(套)，农机总动力达395万千瓦。

——城乡面貌变化显著。坚持以规划为引领，城乡发展一体化步伐加快。开展城市总体规划实施情况评估，专项规划、控制性详规等加快完善，市城区控规覆盖率95 % 。“大十字”城镇群经济社会发展规划、区域规划、土地利用规划和全市县城总体规划修编(修改)以及村镇规划编制进程加快。市城区以“三创”和“创模”为抓手，实施基础设施项目211个，完成投资197.3亿元。改造背街小巷40条，拆除违法建(构)筑物38.5万平方米，45个棚户区、13个城中村和主要街道街景改造项目稳步推进。建成供水设施及配套管网126.9公里，建设(疏浚)污水管网151.5公里。“绿城攻坚”三期任务超额完成，“12234”城市绿化精品建设基本完成，建成区绿化覆盖率、绿地率分别达39.12%和37.1%，人均公共绿地面积10.24平方米。新增天然气公交车187台，城市公交不断改善。投放公共停车泊位3.62万个，其中108个机关企事业单位7000多个停车位对外开放，停车难、乱停车问题有效缓解。启动公共自行车租赁工程，完成公共直饮水工程，城市品质明显提升。数字城管案件结案率97.9%。“创园”、“创模”工作通过验收，“创卫”工作有序推进。“大十字”城镇群列入全省城镇化建设示范点，实施重大项目343个，完成投资584.3亿元，郴永大道全线通车，环市城区半小时城镇圈形成。郴资桂“两型社会”示范带建设规划纲要和综合配套改革总体方案获批，示范带区域土地利用总体规划修编基本完成，346个重大项目完成投资539.4亿元。特色小城镇建设加快发展，示范镇完成投资21亿元，永兴、嘉禾入选国家智慧城市试点单位。创建省级新农村示范村3个，郴州大道沿线初步形成全省首个百里新农村示范带。嘉禾县获评全国农村社区建设全覆盖试验示范县。农村环境整治成效明显，村民建房管理逐步规范。“点亮郴州”行动全面推进，推广LED灯1.8万余盏，公路通道亮化922公里。推进国家森林城市创建，绿化造林72.3万亩，公路通道绿化3667公里，“三年城乡绿化攻坚”任务提前一年超额完成。汝城县获评“全国绿化模范县”。全市森林覆盖率65%。“十大环保工程”加快推进，东江湖水环境保护、市城区清洁能源推广等7项工程完成或超额完成年度任务。扎实开展“水利攻坚年”活动，180座水库除险加固项目和沤菜水库大坝主体工程完工，解决了26.7万农村人口饮水安全问题。获批全国水生态文明城市建设试点。永兴、资兴纳入第五批中央财政小型农田水利重点县。东江湖列入国家重点流域和水资源生态补偿试点、纳入国家重点支持生态湖泊保护范围。农村公路养护全面提质，市郊客运公交化改造稳步推进，建成农村公路713公里、客运站11个，96.8%以上的行政村通客运班车。罗霄山片区县扶贫攻坚加快推进，启动片区规划项目265个、完成投资146.7亿元，桂汝106国道扶贫攻坚示范带建设初见成效。11.3万贫困人口实现脱贫。

——改革开放再创佳绩。持续开展“推进改革年”活动，“8大领域45项关键环节改革”全面实施，企业国资监管在全省率先实现全覆盖;非公经济加快发展，各类市场主体、新增私营企业分别增长22.2%和51%;省级园区行政审批授权基本到位;在全省率先开展城乡居民大病医疗保险试点;郴资桂“两型社会”示范带配套改革、农村土地产权等改革有序推进;省联合产权交易所郴州公司获批，郴州报业文化传媒公司组建。郴州被列为全省两个商事登记制度改革试点市之一。实施承接产业转移“四千工程”，湘南承接产业转移示范区排头兵地位增强。承接园区扩容升级步伐加快，10个省级园区规划面积增加到152平方公里，新增省级工业集中区3个，“一县一园”格局形成。郴州高新区申报国家高新区进展顺利，郴州经开区申报国家经开区工作全面启动，郴州出口加工区进出口总额跃居中部地区出口加工区第一。新建标准厂房375万平方米，出租率95%以上;承接产业转移项目390个。铁路口岸开放，铁海联运开通，湖南至香港直通车运营，郴州迈入“无水港”城市行列。成功承办首届湘南承接产业转移投资贸易洽谈会，省政府明确将以郴州为中心把湘南打造成湖南会展业次中心区域。飞利浦公司入驻，引进世界500强制造企业实现“零”突破。实际利用外资10亿美元、到位内资310亿元，分别增长33.3%和15.3%;外贸进出口总额34亿美元，其中加工贸易26亿美元，分别增长22.3%和36%。

——社会发展更加和谐。“民生100工程”目标任务全面完成，涉及民生的财政支出占总支出的69.7%。新增城镇就业6.7万人、农村劳动力转移就业4.8万人，城镇登记失业率控制在3.96%。社会保障“一卡通”工程建设进展顺利。新农合筹资标准、住院费用实际补偿率和城乡低保对象补助标准进一步提高。新(改)建乡镇敬老院22所，建成社区居家养老服务中心(站)730个、农村幸福院158个。新开工保障性安居工程住房4.96万套(户)。教育强市建设取得新进展，市城区和县城新增义务教育学位2.8万个，创建村级合格学校164所，完成中小学校舍安全工程项目78个。新建乡镇(街道)公办幼儿园20所。建成省级示范性职业学校3所。市特殊教育学校主体工程完工。郴州师范升“专”创建有序推进。湘南学院通过教育部本科教学工作合格评估。郴州职业技术学院、技师学院、广播电视大学稳步发展。国家基本和重大公共卫生服务项目全面完成，基本药物制度实现全覆盖。区域卫生信息化平台建成，市城区社区卫生服务中心标准化建设全面推进。宜章成功创建全国卫生应急示范县。食品药品监管全面加强。文化“双十工程”加快实施，解决广电盲村用户收视问题4万余户。人口计生“基层基础提升年”活动扎实有效，“阳光计生”考评机制在全国推广，人口自然增长率6.27‰。体育之城加快创建，成功举办2013年郴州·中国汽车拉力锦标赛、2014年世界排球锦标赛亚洲区资格赛。新建平价商店44家，物价总体平稳。第三次全国经济普查全面开展。新闻出版、民族宗教、外事侨务、统计、贸促、海关、检验检疫、档案、地震、水文、气象、科普、地方志、爱卫、慈善和工会、妇女、儿童、老龄人、残疾人等事业取得新进步。驻郴部队和国防后备力量为地方发展作出新贡献。公路车辆“双超”率保持0.1%以内。安全生产形势总体平稳。加强社会治理、社区建设和信访工作，深化“打黑除恶”等专项行动，社会安定有序。资兴市获评全国社会管理综合治理“长安杯”。郴州市综治民调满意度位居全省第一。

——政府建设全面加强。认真落实中央“八项规定”和省委、市委“九项规定”，作风大整顿“发展环境整治年”活动取得实效，“电视问政”活动产生广泛影响，发展环境持续优化。市本级第五轮行政审批制度改革完成，审批事项时限整体压缩60.5%。市县两级网上行政审批实现部门、事项、流程全覆盖。行政处罚裁量权基准制度更加健全。综合绩效评估体系进一步完善。财政预决算等8大领域政府信息公开扎实推进。完善市政府领导接听市长公开电话、接待信访群众并督办落实等工作机制，基本建成市县两级社会求助平台，市长公开电话群众来电办结率、群众满意率分别达100%和95%以上。加强审计监督、财政投资评审和预算绩效管理，审计核减项目投资11.8亿元。全市财政专户全部归口国库管理，市本级公务接待费下降22.5%。坚持政府常务会议学法制度，全面实施政府法律顾问制度。认真执行市人大及其常委会决议决定，坚持重大事项向人大报告、向政协通报，自觉接受人大和政协监督，办理市人大代表建议257件、市政协提案267件。充分发挥民主党派、工商联、无党派人士和人民团体参政议政作用。政府公信力和执行力进一步提升。

各位代表!一份耕耘，一份收获。刚刚过去的2013年，我们在“稳增长”中打造了新亮点，在“调结构”中迈出了新步伐，在“促改革”中实现了新突破，在“惠民生”中收获了新业绩。这些成绩的取得，是省委、省政府和市委正确领导的结果，是各级人大、政协监督支持的结果，更凝聚了全市人民的智慧和汗水。在此，我代表市人民政府，向全市各族人民，向人大代表、政协委员，向各民主党派、工商联、无党派人士和人民团体，向驻郴解放军、武警和消防官兵，向中省驻郴单位，向离退休老同志，向所有关心支持郴州发展的各界朋友，表示衷心的感谢并致以崇高的敬意!

回顾一年来的工作，我们也清醒地看到存在的困难和问题：重大项目储备不足，产业结构不优，创新能力不强，产业转型难度和经济下行压力较大;城乡区域统筹发展任务艰巨，居民持续增收难度增加;就业、教育、医疗、住房、生态环境、食品药品安全、社会治安等事关群众切身利益的问题较多，民生改善与群众期盼还有较大差距;行政效率、服务意识有待提升，政风和廉政建设有待进一步加强等。对此，我们将采取有效措施，切实加以解决。

二、2014年工作安排

2014年是“大干新三年、再创新辉煌”的收官之年，也是全面深化改革的开局之年。面对困难和挑战，我们将增强机遇意识，坚持底线思维，强化问题导向，推进改革创新，扎实做好政府工作。根据市委经济工作会议精神，今年政府工作的总体要求是：全面贯彻落实党的十八大和十八届二中、三中全会精神，牢牢把握“稳中求进、改革创新、又好又快”的总基调，按照“四化两型”“三量齐升”的总要求和“一化两城三创四大”的总体思路，全面深化改革，加快转型发展，强化公共服务，突出民生优先，打造郴州经济升级版，实现经济持续健康发展和社会和谐稳定，为全面建成小康社会打下坚实基础。主要预期目标是：地区生产总值增长11.5%左右;财政总收入和公共财政预算收入分别增长15%;固定资产投资增长28%;社会消费品零售总额增长13.5%;城乡居民人均可支配收入分别增长10%和14%;城镇登记失业率控制在4.5%以内;居民消费价格总水平涨幅控制在4%左右;人口自然增长率控制在8.1‰以内;完成节能减排任务。重点抓好以下七个方面：

(一)以改革开放为推动，释放经济社会发展活力。以改革推动发展，以开放激发活力，让一切市场要素的活力竞相迸发，让一切创造社会财富的源泉充分涌流。全面深化各项改革。认真贯彻落实《中共中央关于全面深化改革若干重大问题的决定》，把改革贯穿于经济社会发展的各个领域各个环节，坚决破除各方面体制机制弊端，使市场在资源配置中起决定性作用和更好发挥政府作用。重点围绕推进湘南承接产业转移示范区、郴资桂“两型社会”示范带、“大十字”城镇群建设、城乡统筹发展、罗霄山片区扶贫开发和生态文明建设，积极推进商事登记制度、政府购买服务、投融资体制等改革，加快转变政府职能;积极培育各类市场主体，支持个体私营企业转型升级，加快发展混合所有制经济;积极推进户籍管理、农村产权制度、社会保障、基本公共服务等改革，加快健全城乡一体化发展体制机制;积极推进国有林场管理体制、资源环境价格等改革，健全能源、水、土地节约集约使用制度，加快生态文明制度建设;深化教育综合改革和医药卫生体制改革，推进社会事业改革创新。加强开放平台建设。坚持规划先行、产业集聚、创新驱动、特色发展，深入实施“产城融合”战略，加快完善园区设施、产业和服务配套，健全落实园区用地、用工等保障机制和财政、融资、环境等支持政策，加大简政放权力度，着力增强园区综合承载力和竞争力。力争园区规模以上工业增加值增长16%，占全市规模以上工业比重提高2个百分点以上;郴州高新区创建为国家高新区，郴州出口加工区升级为综合保税区，郴州经开区和资兴经开区创建国家经开区取得突破。整合口岸资源，加快铁海联运三期、郴州口岸国际物流中心和电子口岸等平台建设，开通“五定班列”，构建国际化口岸通关体系。大力承接产业转移。深入实施承接产业转移“四千工程”，主动对接世界500强企业、大型央企、百强民企和上市公司，重点引进一批引领型、补链型、核心型产业项目，抓好签约项目落地建设，大力发展总部经济。加快转变外贸增长方式，提高出口产品技术含量、附加值和品牌竞争力。鼓励市内优势企业参与国际合作，扩大对外投资和劳务输出。建立外贸预警机制，积极应对贸易磨擦。加强区域经济合作。力争新建标准厂房280万平方米;实际利用外资、到位内资均增长15%;外贸进出口总额增长20%，其中加工贸易增长20%。

(二)以扩大内需为抓手，增强经济增长内生动力。抓住宏观经济倒逼扩内需、调结构新机遇，强化项目带动，培育消费热点，着力扩大有效投资和消费需求。把产业投资放在优先位置，深入实施“十二五”投资和项目建设“836”计划，力争完成固定资产投资1883亿元。围绕交通大建设、产业大转型、城市大提质、民生大改善，拟安排总投资1亿元以上主要建设项目920个，年度计划投资1261亿元。其中：基础设施项目231个，年度计划投资321亿元，厦蓉高速公路郴州段、26个干线公路项目400公里全部建成通车，南岭大道南段、郴州汽车总站搬迁等项目竣工，桂新高速公路、城市管道燃气建设等项目加快推进，力争三南高速公路、郴州高铁西站扩建等项目开工建设。产业建设项目322个，年度计划投资457亿元，力争春意昊斯特空调产业链、华信有色三期等项目建成投产，中昆总部经济园一期、富士生态产业园、中湘钨业二期、桂阳功达轮胎、临武正邦养殖及加工、安仁中药材产业化等项目加快推进。节能环保项目75个，年度计划投资94亿元，启动市第二和第四污水处理厂、永兴意水有色金属废料回收利用等项目建设，加快推进湘江流域重金属污染治理、城乡生活垃圾及污水处理等项目。民生和社会发展项目292个，年度计划投资389亿元，启动市一医院东院、市二医院整体搬迁、苏仙太阳城养老社区等项目建设，加快推进市文化艺术中心、临武文化体育广电中心等项目。全面深化银企合作，规范发展融资平台，努力扩大民间投资，创新融资手段和工具，力争各类融资200亿元以上。加强土地调控，强化用地保障。完善重大项目联动协调、督查督办和考评问责机制，严格项目全程质量、安全和工程监理，优化项目建设环境，确保工程进度和质量。强化政府投资项目全程监管和跟踪审计。全面提高城乡居民收入，释放和提升居民消费能力。巩固传统消费市场，着力开拓中介服务、高新技术服务等新领域，大力培育文化、教育、家居服务等新热点，积极发展电子商务、网络购物等新业态。加快推进“市场建设三年行动计划”和“百城千镇县乡流通再造工程”，重点在市城区及郴州大道沿线建设好1个现代农产品批发中心、9个专项市场和7个城市综合体，以及裕后街美食文化街区等特色商业街区，全面完成市城区农贸市场提质改造，改善提升农村消费环境。加快国家再生资源回收体系综合试点城市和农产品现代流通综合试点城市建设。加强产品质量、价格和市场秩序监管，营造安全放心的消费环境。

(三)以产业转型为核心，提升经济发展质量效益。深入推进“产业转型发展三年行动计划”，确保三年目标任务圆满完成。坚持调优结构，提升服务经济。把发展现代服务业作为重大战略，坚持现代服务业与先进制造业融合发展、现代服务业集聚区与开发园区配套建设，以市城区为核心、各县城和中心镇为重点，依托城际大道、国道、高速公路等交通干线，着力构建“一核四极多区外环”的现代服务业发展格局，推进现代服务业发展提速、比重提高、水平提升。加快推进湘南国际物流园、宏进农副产品国际物流中心等物流园区和专业物流中心建设，大力引进和培育现代物流龙头企业，提高物流企业市场竞争力。积极发展物联网产业，培育和扶持发展一批物联网骨干企业。全面实施旅游新“五个一”工程，开展“中国温泉之城建设攻坚年”活动，办好2014年中国(郴州)国际休闲旅游文化节。积极推进苏仙岭、万华岩、龙女温泉等景区打捆创建国家5A级景区。加快飞天山文化旅游创意园、中国银都文化产业园等建设，促进文化与旅游融合发展。扎实创建金融生态良好城市，加强股份制银行入郴工作，推进县域农信社和农村金融综合改革，引导金融机构向农村和社区延伸营业网点，确保郴州农村商业银行上半年挂牌。规范发展小额贷款、融资性担保公司，探索成立小额再贷款公司。金融机构新增贷款110亿元。强力推进企业上市，确保金贵银业成功上市。推进信息基础设施改造升级，加快“数字郴州·智慧城市”建设。加快服务外包平台建设和主体培育，积极创建中国服务外包示范城市。大力发展健康服务业，鼓励发展家政服务业，突出发展养老服务业。力争服务业增加值增长12%。突出“两型”引领，提升工业经济。加快有色金属“五个一”战略体系建设，大力发展精深加工业，重点支持国家有色贵重金属郴州质检中心、世界有色金属博物馆等服务平台建设，加快推进桂阳有色金属冶炼加工项目区建设，力推有色金属产业转型升级。重点支持高斯贝尔、台达电子、华磊光电、飞利浦照明、临武东佳电子等优势企业发展，大力发展消费类整机、光电子、电子材料、电子元器件等四大电子信息产业集群，打造全国一流的LED产业基地和千亿产业集群。积极支持格兰博、奥美森、伯朗电梯等优势企业发展，加快发展先进制造业。重点支持中化弘源化工、中盐华湘等骨干企业技术改造，加快氟化工产业基地建设。重点支持郴州烟叶复烤厂搬迁、郴州烟厂异地技改等项目建设，推进烟叶生产及深加工基地建设。重点支持南方石墨、明大石墨(碳素)制品加工等企业和项目发展，积极发展新型建材。重点支持风电、生物质能发电、腾翔新能源等项目建设，推进新能源产业发展。积极稳妥推进落后小煤矿关闭退出，促进煤炭企业集团化。实施企业信息化“登高计划”，加快推进公共服务领域信息化。力争工业技改投资增长23%，战略性新兴产业增加值增长20%，规模以上工业增加值增长13.5%。围绕农民增收，提升农业经济。加强基本农田保护和抛荒耕地治理，建设高标准农田7.8万亩，粮食总产量稳定在190万吨以上。扎实推进“菜篮子”工程，积极创建国省级畜禽养殖标准化示范场和水产健康养殖示范场，种植蔬菜145万亩。优化农业产业布局，突出抓好以郴桂嘉为重点的现代烟草农业示范区和郴资桂-郴永宜现代农业示范带建设，创建11个现代农业示范园区，收购烟叶106万担。加强标准化茶园生产基地建设，新增种植面积5万亩。推广农业标准化生产面积260万亩，农产品农残检测合格率97%以上。大力推进农业产业化“12345”工程，加快发展农林产品精深加工业，重点支持临武城东、桂阳芙蓉、资兴罗围等农产品加工园区建设，重点扶持一批农业龙头企业、农民专业合作社、农村专业服务组织和种养大户、家庭农场。力争新增500家农民专业合作社、1家国家级农业龙头企业，培育年产值过10亿元龙头企业1家、过5亿元4家、过亿元40家以上，农产品加工业产值增长21%。积极引导和规范农村土地流转，大力发展庄园经济和休闲农业。每个县市区建成1个高标准现代精品农庄，市里重点抓好郴州大道沿线现代休闲农庄的规划建设。推广新型农机具5.7万台(套)，新增农机总动力10万千瓦。做好防汛抗旱、动植物防疫等工作，抓好农房等涉农保险。推进产学研结合，提升创新能力。实施一批重大科技专项，引导企业与高校、科研院所共建技术创新平台，着力破解重点产业领域的关键共性技术。积极推进市国家级示范生产力促进中心、中南大学技术转移中心等科学创新平台，以及永兴国家稀贵金属再生利用产业化基地建设。重点培育首批10家科技创新型企业，力争组建2家科技公共服务孵化器。加强知识产权保护和运用，强化激励，用好人才。实施商标品牌战略，引导企业开展国际商标注册。力争高新技术产业增加值占GDP比重提高2个百分点，新认定国家高新技术企业20家，专利授权突破1000件。

(四)以城镇群建设为龙头，推进城乡区域统筹发展。以新型城镇化为引领，强化项目带动和产业支撑，以农业转移人口市民化为首要任务，增强“大十字”城镇群发展极核和辐射带动作用，促进城乡发展一体化。强化城乡规划引领。全面完成城市总体规划实施评估工作并启动总规修改工作，基本完成城市综合交通、排水防涝、历史文化名城保护等专项规划编制，力争市城区控规覆盖率98%以上。推进县市城市总体规划修编(修改)，全面完成镇乡域村镇布局规划编制。严格规划管理，维护规划权威。做强做优中心城区。深入推进“十大提质”，全面实施“五化工程”，加快新城区建设和下湄桥片区、棚户区、城中村改造提质步伐。拟安排城市基础设施项目227个，年度计划投资196亿元，着力完善城市基础配套、交通配套、生活配套和生态景观系统，不断增强城市综合承载能力。实施东江引水工程，改造万华、山河水厂和城市供水管网，力争2015年市城区公共供水普及率达到95%。开展城市交通秩序、市容市貌、卫生保洁等专项整治行动，开展垃圾分类收集试点，加快推进管护作业市场化，促进城市管理数字化、规范化和市场化。大力发展城市公交，合理规划设置车行道、人行道和停车位，加快武广高铁等6个公交场站和公共自行车租赁工程一期建设，缓解道路拥堵和停车难现象。巩固“创园”、“创模”成果，扎实推进“创文”工作，力争成功创建国家卫生城市。加快发展“大十字”城镇群。完成“大十字”城镇群经济社会发展规划、区域规划、土地利用规划以及各项重大专项规划编制，并组织实施。实施城镇群“交通同网、城镇同建、产业同兴、环境同治、城乡同筹”五大工程，加快区域内343个投资过亿元重大项目建设，担当起全省新型城镇化试点重任。加快资兴、桂阳、永兴、宜章融城步伐。力争进入国家中小城镇群建设试点。做大做特县城和小城镇。以人为核心，推进县城扩容提质，完善配套设施和公共服务，做实产业基础，提升县城中心地位。开展“美丽宜居村镇”创建活动，以30个示范镇为重点，扩大镇级管理权限，加快完善供水、供气、排污、垃圾处理等公共基础配套和公共管理、公共服务，不断增强对周边村庄的吸纳带动力。启动资兴市等8个自来水厂建设改造项目，力争2015年全市县城公共供水普及率达到85%，所有示范镇实现污水配套管网全覆盖。大力发展小城镇特色产业，培育一批工矿型、商贸型、现代农业型、生态旅游型等特色鲜明的小城镇。把县市区作为全面小康建设的主体，完善差别化工作思路、支持政策和考核办法，促进县域经济加快发展。积极推进新农村建设。坚持历史保护、生态建设、产业发展相结合，以郴州大道新农村建设示范带为龙头，推进“十城百镇千村”新农村建设工程。每个县市区高标准打造1-2个新农村示范区，抓好1个试点镇连片带动5个以上行政村的新农村建设工作。积极开展“美丽乡村”创建活动，持续推进“点亮郴州”行动，大力实施以“四改、四清、两绿、一集中”为重点的农村环境整治，改善农村人居环境。重点抓好乡镇政府驻地、主要景区和交通沿线、城乡结合部村庄的环境整治，力争40%的行政村达到“三无三有”目标，新增省级美丽村庄5个以上。加强农村土地和规划管理，坚持适度集中、一户一宅、拆旧建新，严格规范村民建房。深入开展农村公路养护大提质。加大扶贫攻坚力度。落实扶持政策，加大资金投入，强化产业培育和公共服务，在4个片区县实施项目280个，力争投资320亿元。继续抓好桂汝106国道扶贫攻坚示范带建设，统筹推进其他县市区200个重点贫困村扶贫工作。加强大中型水库移民避险解困工作，重点扶持少数民族和移民地区加快发展。力争12万贫困人口脱贫。

(五)以绿色发展为引领，全面加强生态文明建设。大力实施“青山、碧水、净土、蓝天”四大工程，争创全国生态文明示范市。全力创建国家森林城市。加大造林绿化和封山育林力度，深入推进城乡绿化攻坚，突出建设以“大十字”城镇群为核心的森林生态“绿心”，构建城乡一体的绿道网络。市城区启动国家生态园林城市创建，加快推进“绿城攻坚”四期，全面开展城市绿荫行动，力争新增城区绿化面积89.8万平方米，种植绿化大苗28.5万株，新建公(游)园17个。力争全市荒山荒地和迹地更新造林40万亩、公路通道绿化1000公里，成功创建国家森林城市。积极推进水生态文明建设。围绕创建全国水生态文明试点城市，扎实开展“水利跨越年”活动，加快实施六大水生态文明工程。高标准推进东江湖保护和开发。力争水利建设投资50亿元，完成病险水库除险加固62座，新修加固堤防268.5公里，治理水土流失面积38.6平方公里，改善灌溉面积34.9万亩，新增村镇供水受益31.5万人。强力推进十大环保工程。加快实施湘江流域郴州段重金属污染治理项目。扎实推进以三十六湾、鲁荷矿区等工矿区以及56座尾矿库为重点的矿山综合治理，以翠江、武水河等为重点的流域水环境集中整治。实施大气污染防治行动计划。加强固体废弃物安全处置和综合利用。强化土壤环境保护和综合治理。大力推进节能减排。加快淘汰落后产能，严格控制高耗能、高污染和资源性行业项目准入。全面推进工业企业、交通运输、公共机构、建筑等各领域的节能节水节地节材工作，引导城乡居民使用节能型产品。加强对重点行业和重点工程减排监管，健全和规范污染减排制度，削减主要污染物排放总量。鼓励企业大力发展循环经济。市城区燃煤锅炉和窑炉全部退出，新增天然气公交车100台。农村推广沼气池3000口、太阳能热水器6000台、高效生物质炉灶6000台。加快推进“两型社会”示范带建设。认真落实示范带建设规划纲要和综合配套改革总体方案，积极探索“两型”建设新模式。大力实施示范带三年行动计划“十大工程”，加快“两型”项目建设，培育“两型”产业，推进配套改革，提升极核地位。

(六)以改善民生为根本，建设平安和谐幸福郴州。坚持民生优先，实施“民生100工程”，让发展成果更多更公平惠及全体人民。持续推进创业带动就业。实施更加积极的就业政策，消除影响平等就业的制度障碍和就业歧视，完善落实扶持政策，降低创业门槛，鼓励自主创业，充分发挥创业带动就业的倍增效应。健全城乡均等的公共就业服务体系，加强职业技能培训和就业援助，统筹做好以高校毕业生为重点的青年就业和农村转移劳动力、城镇困难人员、退役军人、化解产能过剩中出现的下岗人员、有劳动能力的残疾人就业。力争新增城镇就业6万人、农村劳动力转移就业3.5万人，城镇“零就业”家庭动态清零，职业技能培训10万人次。着力提高社会保障水平。建立健全城乡一体的社会保障制度，统筹推进城乡最低生活保障制度和城镇企业基本养老、城乡居民大病医疗保险，稳步推进新型农村社会养老保险、建筑行业农民工工伤保险。力争城镇职工基本医疗保险、城乡(城镇)居民医疗保险和生育保险参保率、新农合参合率稳定在95%以上。加快发展养老服务业，大力推进社区居家养老服务中心(站)、农村幸福院建设，争创“全国养老服务示范城市”。支持社会慈善、社会捐助、社会福利等事业发展，完善社会救助体系，争创“全国慈善城市”。健全住房保障、供应和后续管理体系，新建(筹建)保障性安居工程住房3.95万套(户)。促进社会事业全面发展。全面落实教育优先发展战略，继续做好市城区和县城义务教育学校扩容、村级合格学校建设、农村薄弱学校改造工作，扩大优质教育资源覆盖面，逐步缩小区域、城乡、校际差距。鼓励发展学前教育、职业教育和民办教育。力争郴州师范成功升“专”。支持湘南学院、郴州职业技术学院、郴州技师学院、郴州广播电视大学发展。强化基本公共卫生服务，巩固完善基本药物制度，提高群众医疗保障受益水平。深入推进乡村卫生一体化管理和市城区社区卫生服务中心规范化建设。做好重大疾病防控和健康教育活动工作。扶持中医药事业加快发展。强化食品药品安全监管，保障食品药品安全。完善公共文化基础设施和服务体系，加快推进文化“双十”工程，丰富群众文化生活，提高文化产业竞争力，塑造郴州时代风尚。积极申报国家级历史文化名城。广泛开展群众体育活动，加快创建体育之城。坚持计划生育基本国策，加强人口计生基层基础工作，促进人口长期均衡发展。支持工会、共青团、妇联等团体组织发挥桥梁纽带作用。维护妇女、儿童、老龄人、残疾人合法权益。抓好第三次全国经济普查。做好广播影视、新闻出版、民族宗教、外事侨务、统计、贸促、爱卫、海关、检验检疫、档案、科普、地方志、水文、气象等工作。加强和创新社会治理。坚持源头、系统、综合、依法治理，改进社会治理方式，激发社会组织活力，提高社会治理水平。高度重视城乡社区建设，完善和创新流动人口、特殊人群服务治理，建立健全基层社会治理服务体系。做好第九次村委会和村务监督委员会换届工作。严格落实安全生产责任制和责任追究制，严防重特大事故发生。继续推进公路“治超”和客运市场整治。深入推进“平安郴州”、“法治郴州”建设，完善立体化社会治安防控体系，依法防范和惩治违法犯罪活动。创新群众诉求处理方式，着力解决信访突出问题和社会纠纷。强化网络社会和公共应急管理，提高预防和处置突发事件能力。加强国防动员、国防教育、民兵预备役、人民防空和国家安全人民防线建设，推动军民融合深度发展，争创“全省双拥模范城”。

(七)以转变职能为重点，打造法治政府和服务型政府。做好今年各项工作，必须以开展党的群众路线教育实践活动为契机，以改革创新精神加强政府自身建设，不断增强政府公信力和执行力。加快转变政府职能。大力简政放权，强化政府公共服务、市场监管、社会治理、环境保护等职能。完善和落实依法科学民主决策制度，规范政府行政行为，提高政府决策水平。深化行政审批制度改革，没有法定依据的审批事项一律取消，中央和省政府明令取消的一律取消，重复审批的一律归并或取消，暂时保留的一律规范提速，并更加注重事中事后监管。推进机构编制管理科学化、规范化和法制化。大力提升行政效能。创新服务方式和理念，有序推进网上政务服务和电子监察平台建设与应用，持续开展“电视问政”。强化行政监察，严格行政问责。完善综合绩效管理与全面小康建设考核相结合的考核评价机制。健全改进作风常态化制度，着力解决群众反映强烈的突出问题。加强公务员队伍建设，提升管理能力、服务能力和执行能力。全面推进依法行政。强化法治思维，践行法治理念，自觉把政府行为置于法律约束之下。严格执行人大及其常委会的决议决定，自觉接受法律和工作监督，主动接受政协民主监督，认真办理人大代表议案、建议和政协提案。广泛听取民主党派、工商联、无党派人士和人民团体意见，虚心接受新闻媒体和社会公众监督。加强行政监察和审计监督。推行政府及其部门权力清单制度，完善政务公开和新闻发布制度，确保权力在阳光下运行。努力建设清廉政府。坚持用制度管权管事管人，健全惩治和预防腐败体系。严格执行党风廉政建设责任制和廉洁从政各项规定，认真执行党政机关厉行节约反对浪费条例，全面停止新建楼堂馆所，全面清理规范公务用车、办公用房，全面规范公务接待。市本级“三公”经费支出原则下降20%。加强预算编制和政府债务管理，健全全程动态监控机制，完善严格的财务预算、核准和审计制度，强化财政资金使用、重大项目投资、工程招投标、政府采购、国有资产资源经营管理等重点领域监管。对腐败分子坚决依法依纪查处，决不姑息纵容!

各位代表!郴州正处在科学发展、加速起飞的关键时期。时代赋予使命，实干铸就辉煌。让我们高举中国特色社会主义伟大旗帜，在中共郴州市委的坚强领导下，团结和依靠全市人民，解放思想、改革创新，凝聚力量、积极作为，用我们的勤劳和智慧，共同创造郴州更加美好的未来!
